# Methods for randomized, blinded, controlled evaluation of putative disease interventions in multilaboratory, preclinical assessment networks

**DOI:** 10.1038/s41684-026-01683-z

**Published:** 2026-02-18

**Authors:** Jessica Lamb, Karisma Nagarkatti, Marcio A. Diniz, Ryan Cabeen, Monica Estrada, Karen L. Crawford, Andre Rogatko, Sungjin Kim, Cenk Ayata, David C. Hess, Mohammad Badruzzaman Khan, Rakesh B. Patel, Mariia Kumskova, Enrique C. Leira, Anil K. Chauhan, SPAN Consortium, Kazi Akhter, Ken Arai, Ali Arbab, Jaroslaw Aronowski, Brooklyn Avery, Hannah Beatty, Adnan Bibic, Frank Blixt, Ligia Boisserand, Marian Cabrera-Ayala, Suyi Cao, Anjali Chauhan, Valina Dawson, Kris Dhandapani, Nirav Dhanesha, Sebastian Diaz, Taylan Erodogan, Andrew Goh, Ali Herman, Shuning Huang, Fahmeed Hyder, Takahiko Imai, Emma Imakavar, Xuyan Jin, Conor Johnson, Pradip Kamat, Senthilkumar Karuppagounder, Raymond Koehler, Ewa Kulikowicz, Javier Labastida, Steven Lannon, Siyue Li, Eng Lo, Joe Mandeville, Michael Maniskas, Louise McCullough, Andreia Morais, Diego Motales-Scheihing, Steward Niefert, Mohammad Nisar, Lidiya Obertas, Tao Qin, Basavaraju G. Sanganahalli, Lauren Sansing, Yanrong Shi, Shahneela Siddiqui, Cameron Smith, Guanghua Sun, Brijesh Sutariya, W. Taylor Kimberly, Dan Thedens, Shun-Mug Ting, Klaus van Leyen, Peter Van Zijl, Sofia Velazquez, Jun Wang, Nicholas Wilder, Kristofer Wood, Jiadi Xu, Lili Yu, Steve Zeiler, Patrick Lyden

**Affiliations:** 1 Department of Physiology and Neuroscience of the Zilkha Neurogenetic Institute of the Keck School of Medicine, Los Angeles, CA USA; 2 Biostatistics and Bioinformatics Research Center, Samuel Oschin Comprehensive Cancer Center, Cedars-Sinai Medical Center, Los Angeles, CA USA; 3 USC Stevens Neuroimaging and Informatics Institute, Los Angeles, CA USA; 4 Neurovascular Research Unit, Department of Neurology, Harvard Medical School, Massachusetts General Hospital, Boston, MA USA; 5 Department of Radiology, Harvard Medical School, Massachusetts General Hospital, Charlestown, MA USA; 6 Department of Neurology, Medical College of Georgia, Augusta University, Augusta, GA USA; 7 Department of Internal Medicine, Division of Hematology, Oncology and Blood and Marrow Transplantation, Carver College of Medicine, University of Iowa, Iowa City, IA USA; 8 Department of Neurology, Carver College of Medicine, University of Iowa, Iowa City, IA USA; 9 Department of Neurosurgery, Carver College of Medicine, University of Iowa, Iowa City, IA USA; 10 Department of Epidemiology, College of Public Health, University of Iowa, Iowa City, IA USA; 11 Department of Neurology of the Keck School of Medicine, Los Angeles, CA USA; 12 Department of Radiology, Johns Hopkins University, Baltimore, MD USA; 13 Department of Neurology, Johns Hopkins University, Baltimore, MD USA; 14 Biochemistry and Molecular Biology, Medical College of Georgia, Augusta University, Augusta, GA USA; 15 Department of Neurology, McGovern Medical School, University of Texas HSC, Houston, TX USA; 16 Department of Neurology, Yale University School of Medicine, New Haven, CT USA; 17 Department of Neurology, Institute of Cell Engineering, Johns Hopkins University, Baltimore, MD USA; 18 Neurosurgery, Medical College of Georgia, Augusta University, Augusta, GA USA; 19 Department of Immunobiology, Yale University School of Medicine, New Haven, CT USA; 20 Department of Biomedical Engineering, Yale University, New Haven, CT USA; 21 Department of Anesthesiology and Critical Care Medicine, Johns Hopkins University, Baltimore, MD USA; 22 Department of Immunology, MD Anderson Cancer Center, Houston, TX USA; 23 Department of Radiology and Biomedical Imaging, Yale University, New Haven, CT USA; 24 Center for Genomic Medicine and Division of Neurocritical Care, Department of Neurology, Massachusetts General Hospital, Boston, MA USA; 25 Radiology, College of Public Health, University of Iowa, Iowa City, IA USA; 26 Present Address: Department of Population Health Science and Policy, Icahn School of Medicine at Mount Sinai, New York, NY USA

**Keywords:** Research data, High-throughput screening, Research management

## Abstract

Science faces a reproducibility crisis, and public trust in science declines when large clinical trials, which had been qualified by promising preclinical studies, fail. While some clinical trial designs may have been inadequate, preclinical assessments of disease interventions might have lacked key elements of rigor such as treatment concealment, randomization, blinded outcomes, prespecified and adequate sample sizes, and models including comorbidities. Here, to demonstrate feasibility and practicality of enhanced rigor in preclinical assessment, we designed a six-laboratory network that implemented rigorous study elements, using acute ischemic stroke for demonstration. This network enrolled 2,615 rodents in 5 different models and implemented a multistage, multiarm statistical design that sequentially eliminated candidate interventions during interim analyses. The methods included centralized intervention packaging, randomization, data quality assessment and data archiving. Blinded analysis of 9,274 video-recorded behavioral tasks and 3,652 magnetic resonance images were evaluated. All tools and protocols are presented and could be adapted to preclinical assessment in other disease areas.

## Abstract (web summary)

This Article details the methods used for rigorous assessment of disease interventions in a multicenter preclinical trial. Using stroke models as proof of concept, it offers adaptable protocols for any field requiring robust preclinical validation.

## Main

Science faces skepticism from the lay public, and scientists have described problems with rigor, transparency and reproducibility. Many published findings, selected from high-impact journals, have failed replication outside of the original laboratories^1–3^. Many factors contribute to reproducibility issues in science: inadequate sample size and proper power analysis before initiating experiments, lack of control for repeated significance testing (‘*P*-hacking’), inadequate blinding of the investigators or insufficient or inappropriate controls, among other deficiencies^1,4–7^. Many groups, including the National Academy of Science, have called on grant agencies and journals to enforce higher standards of rigor and experimental design to address these deficiencies. However, appropriate methods to implement greater scientific rigor may be lacking or insufficiently developed^8^.

Here, we address one important type of scientific study: the use of preclinical animal disease models to assess the efficacy of proposed candidate interventions. Before launching pivotal clinical trials in patients, many funders, sponsors and regulators require that therapeutic efficacy be documented in an accepted animal disease model. Typically, such animal disease models replicate some key aspects of human disease; it is assumed that results from the animal disease model anticipate the results of subsequent human clinical trials. Too often, however, promising interventions—despite positive results in preclinical studies using animal disease models—fail to translate into clinical trials. This was recently observed with a widely touted antibody targeting cerebral β-amyloid in patients with Alzheimer’s disease, for example^9^. Similar failures have been noted in neuroscience, cardiology and oncology, among other areas^2,3,10,11^. Although the failures in clinical translation may partly result from an inadequate design of the clinical trial, our concern here is to improve the quality and validity of the preclinical assessment of candidate interventions.

Key elements of design quality in preclinical assessments include treatment concealment during disease induction, participant randomization, blinded outcome assessment and adequate, prespecified sample size^5,12–14^. These key elements may be challenging to address in a single laboratory if too few personnel are available to separate the randomization process from the treatments and assessments to maintain blinding. Even if the steps can be separately tasked, the interventions under study often can be identified if they look different or are dosed differently. Study outcome variables, such as behavior, histomorphometry and image analysis, should be assessed by a completely independent investigator unaware of treatment grouping. All these processes must be performed simultaneously yet independently, requiring yet another entity to combine all input into a common file so that data can be analyzed, also in a blinded manner. Historically, all these steps do not occur in most academic or contract laboratories owing to a lack of funds to employ so many independent workers.

We sought to create and organize the operational methods needed to conduct an effective, rigorous and successful preclinical assessment of putative disease interventions^15,16^. Our study was a large preclinical, multicenter trial and demonstrated the feasibility of all essential elements of rigor and data transparency. Our intention was that our methods could be used in any field that requires rigorous preclinical demonstration of treatment efficacy in an animal disease model. As proof of concept, we conducted a study of six putative cerebroprotective interventions for acute ischemic stroke, an area in which failure to translate positive preclinical assessments into clinical trial success has been well documented^10,17–19^. While the overall implementation and results of our stroke-specific preclinical network have been already published^20–22^, here we present the specific operational methods in much more detail to enable other groups seeking to accomplish preclinical assessments in other specialties or fields with the same utmost rigor. Further details and specific protocols are provided in the Supplementary Information.

## Results

### Initial framework and approach

**Fig. 1:**
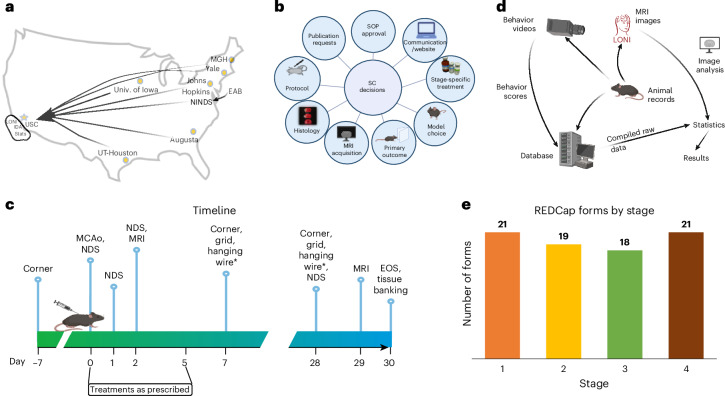
Description of the network. **a**, Geographical representation of the six laboratories marked with a yellow dot (Augusta, University of Iowa, Johns Hopkins, Mass General Hospital (MGH), Yale and UT-Houston) sending data to the CC at University of Southern California (USC), marked with a yellow star, where the data repositories are located, including IDA of LONI and Statistics. The External Advisory Board (EAB) provides feedback to the National Institute of Neurological Disorders and Stroke (NINDS) in Washington DC, which also advises the network. **b**, Graphical representation of the decisions the SC makes, including approving SOPs, communication and website development, stage-specific treatment protocols, the model choice, behavioral outcome measures and the experimental protocol, which includes when MRI images are collected as well as histology decisions and publication requests from within the network and outside the network. **c**, General experimental timeline through end of study (EOS): each animal starts with baseline corner testing performed seven days before MCAo surgery. NDS are collected on the day of surgery, day 1, day 2 and day 28. MRI is performed at day 2 and day 29. In addition to the baseline, corner testing is performed at day 7 and day 28, along with grid testing and hanging wire testing. *The hanging wire test was discontinued after stage 1. **d**, Pathways of data flow from the research laboratories to central storage and analysis. Data collected at the laboratories included animal records, MRI images and behavior videos, which are sent to either other laboratories, the database or LONI for analysis. Once compiled, all raw data are sent to statistics for results. **e**, Total number of data entry forms in the REDCap database for each stage (1–4) of the trial. Panels **a**–**d** created in BioRender; Lamb, J. https://biorender.com/sae4s8q (2026).

The Stroke Preclinical Assessment Network (SPAN) was funded by the National Institute of Health (NIH) and included a coordinating center (CC) and six US research laboratories (Fig. 1a). The participating SPAN research laboratories and the six study interventions were selected via peer review^20^; in other applications, the collaborating laboratories might be selected through peer review or may self-select as part of a voluntary collaboration. To provide central coordination, a laboratory with prior experience in both preclinical modeling and managing multicenter clinical trials was selected as the CC through peer review. During SPAN, the CC was not directly involved in performing the animal disease model or generating any of the outcome data; instead, the CC served as the central data depot, managed intervention supply, randomized interventions, assigned digitally recorded behavioral assessments for blinded review and performed all statistical analysis. The study Steering Committee (SC) was composed of the Principal Investigators (PIs) from each research laboratory, included NIH representatives, and was chaired by the CC PI.

To simultaneously evaluate six intravenous (IV) and intraperitoneal (IP) interventions in parallel, each tested against an appropriate control, SPAN used a multiarm, multistage (MAMS) design, using four sequential stages^23–25^. At the end of stages 1–3, interim analyses were performed: study interventions that seemed futile or efficacious were to be dropped from continued testing using pre-specified futility and efficacy criteria. At the end of stage 1, none of the interventions was dropped; after stage 2, three interventions were dropped; after stage 3, two more interventions were dropped, leaving one intervention for final testing in stage 4. At the end of stage 4, a final analysis that included all data from all stages confirmed that the one remaining intervention exceeded the prespecified boundary for declaring it efficacious.

Over several months, the SC debated and finalized standard operating procedures (SOPs) for every method or course of action^26^, including the selection of behavioral and imaging endpoints suitable for the chosen disease model (Fig. 1b). The CC initiated the trial with an in-person kick-off meeting. The SC approved 57 laboratory-specific SOPs and an additional 7 CC-specific SOPs (available by contacting spancc@usc.edu)^20,22^, although not all SOPs were completed before initiating the trial. Initially, the network envisioned using young, healthy mice for the four stages of the trial. However, after the stage 1 interim analysis, the SC, aiming to better replicate human conditions, voted to include aged mice, obesity-induced mice and spontaneously hypertensive rats in stages 2 and 3 and healthy, young Sprague-Dawley rats in stage 4. Although SOPs related to surgery, treatment administration and behavioral assessments were created before the start of the trial to ensure that all necessary protocols were in place for the trial’s initiation, SOPs for comorbid conditions were developed before stage 2. In collaboration with the network, the CC created SOPs outlining the feeding schedule, blood glucose monitoring in diet-induced obese mice, procedures for ordering animals from the National Institute on Aging (NIA) and protocols for requesting blood pressure readings from The Jackson Laboratory before delivery to site. The adoption of SOPs was intended to standardize procedures across the network, but the investigators recognized the importance of embracing heterogeneity when attempting to model human disease in animal models. Human stroke trials are characterized by considerable heterogeneity that is often ignored in preclinical modeling. A combination of mice and rats was used in SPAN to leverage the strengths of each species and to encompass a range of models that reflect diverse stroke pathophysiologies. A stroke model was used that involved temporary occlusion of the middle cerebral artery (MCAo) in equal numbers of males and females in five different animal models: normal young adult mice, aging mice (15–17 months), mice with diet-induced obesity and hyperglycemia, normal young adult rats and spontaneously hypertensive rats.

Subject randomization was stratified by research laboratory, sex and type of stroke model. After the randomized intervention assignment, subjects underwent stroke surgery and received their assigned intervention, followed by behavioral and imaging assessments (Fig. 1c). To obtain early indications of outcomes, we obtained brain lesion morphometry with magnetic resonance imaging (MRI) at 48 h, and neurological deficit scores (NDS) at 24 and 48 h. A corner test was performed after 7 days to have some outcome data if the animal did not survive to the end of the study. To assess the long-term effects of stroke treatment, we performed a final corner test and MRI scan 30 days after treatment. Human clinical trials require functional outcomes, such as disability ranking or neurological deficit scales, so we designated the 30-day corner test as the primary outcome. Treatment-specific details such as dose and timing have been published. IV interventions were administered only on the day of surgery, whereas IP interventions were administered on the day of surgery and then for 3 days after surgery. Dosing (sex and species) was determined by the research laboratory that proposed the intervention^22^. Digital video recordings of the behavioral assessments and the magnetic resonance (MR) images were uploaded to the Imaging Digital Archive (IDA) of the Laboratory of Neuroimaging (LONI)^27^ for blinded review and quantification (Fig. 1d). Drug intervention vials were shipped as needed to the laboratories before randomization. The number of unique data-collection forms was relatively constant per stage (Fig. 1e), allowing the four data stages to be combined for statistical analysis.

### Cohort control and subject flow

**Fig. 2:**
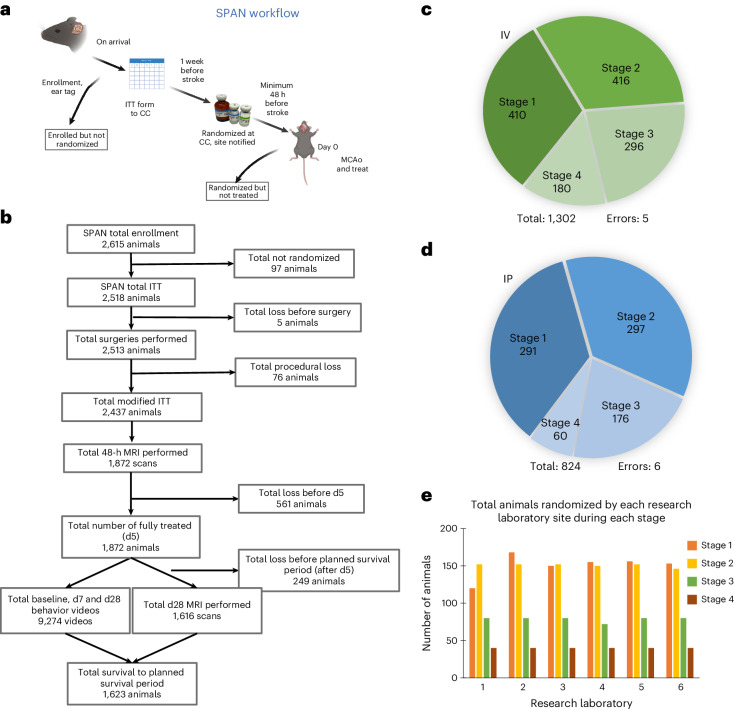
Subject flow and disposition. **a**, Workflow for the randomization process for every animal enrolled. After enrollment and ear tagging upon arrival at the site laboratory, an ITT is completed and submitted to the CC a week before the scheduled MCAo surgery. Once randomized, the site is notified via email a minimum of 48 h before surgery, which is considered day 0. Any animal not randomized or randomized and not treated is still accounted for in cohort descriptions. **b**, Population group sizes following CONSORT guidelines. For transparency, every animal ordered, purchased or bred for the purpose of inclusion in the SPAN study is accounted for in each cohort. **c**, Total animals for each stage that received IV interventions and total medication errors for all four stages combined. **d**, Total animals for each stage that received IP interventions and total medication errors for all four stages combined. **e**, Total animals randomized during each stage at each of the six research laboratories. Panel **a** created in BioRender; Lamb, J. https://biorender.com/sae4s8q (2026).

Attrition bias results when investigators control and censor individual subjects after randomization and intervention^28^. In clinical trials, attrition bias is managed with intention-to-treat (ITT) analysis: patients are grouped into cohorts as randomized, not as actually treated, and dropout (that is, lost-to-follow up) patients remain in the ITT analysis^29^. Cohort and subject control have not previously been addressed in preclinical trials, and we implemented a workflow to account for every subject (Fig. 2a). To prevent investigator’s influence over subject cohorts, research laboratories assigned a unique subject identifier and attached an MRI-compatible, bar-coded ear tag (Supplementary Fig. 1) to each study subject upon arrival. Ear tags were to remain affixed throughout all study procedures. After randomized assignments were sent to the research laboratories, surgery to induce stroke was performed and the assigned intervention was administered. The ITT analysis population was defined as all subjects that were randomized (Fig. 2b). The modified ITT analysis included subjects that completed the disease model surgery and began the intervention^30^. The per-protocol (PP) analysis included subjects who completed all assigned intervention doses and survived 5 days after surgery (Fig. 2b). Throughout the follow-up period, subject dropout occurred due to death. We used the Consolidated Standards of Reporting Trials (CONSORT) approach (Fig. 2b) typically required in clinical trials to account for all subjects in this study. The CONSORT approach is used to account for all subjects and to avoid bias resulting from subject exclusions or data censoring^31^. Correct intervention assignment—both in route and specific intervention—was confirmed post hoc and performed well for both IV (Fig. 2c) and IP (Fig. 2d) interventions. The stratified randomization process worked as intended: equal numbers of subjects were enrolled at each research laboratory across all treatment and animal model groups (Fig. 2e). These data confirm that the methods devised for stratified randomization across multiple research laboratories and stages are feasible and worked well. These data may guide the planning of future networks in other disease areas.

### Concealment and blinding

**Fig. 3:**
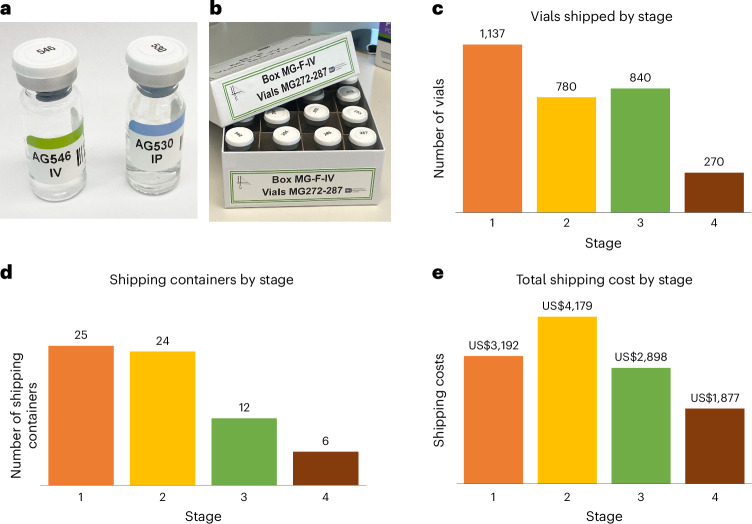
Intervention concealment and supply to research laboratories. **a**, Example of labeled vials. **b**, Example set of IV vials ready to be shipped to research laboratory. **c**, Total number of vials shipped to research laboratories for each stage. Each subject received treatment from one vial. **d**, Total Thermos-controlled shipping boxes/containers shipped to research laboratories for each stage. This number indicates the total number of shipments from the CC to the laboratories. **e**, Total cost of shipping for each stage, which declined due to a combination of efficiency and diminishing sample size as some treatments dropped.

To avoid bias when developing the animal disease model, it is essential to conceal the group assignment from the investigator generating the disease model, for example, performing the stroke surgery. Given that at most laboratories the surgeon also administered treatments, it was essential that treatment identity was concealed. This prevents the surgeon from subconsciously biasing one or another treatment group. Because many putative treatments look different (Supplementary Table 1) and could easily be identified, the challenge in this multiarm, multilaboratory trial was to conceal the identity of the interventions by packaging them identically. In SPAN, all drug interventions were prepared in identical appearing vials (Fig. 3a, Supplementary Note 1, Supplementary Fig. 1 and Supplementary Table 2) and packed in coded, labeled vial boxes (Fig. 3b). The vial boxes were arranged identically across the research laboratories to simplify preparation at the CC (Supplementary Fig. 2). Vial box loading was confirmed independently by two investigators at the CC to assure correct loading. After packaging, vial boxes were shipped in thermoprotected containers with a temperature excursion monitor. Owing to the multistage approach, in which some study interventions may be dropped, the number of shipped vials (Fig. 3c) and the number of shipping containers (Fig. 3d) decreased over time. Reduced sample size per stage and improved efficiencies allowed the total cost of shipping (Fig. 3e) to decrease over time. These data may help in planning future preclinical networks.

### Assessing behavioral outcomes

See also Supplementary Note 2.

**Fig. 4:**
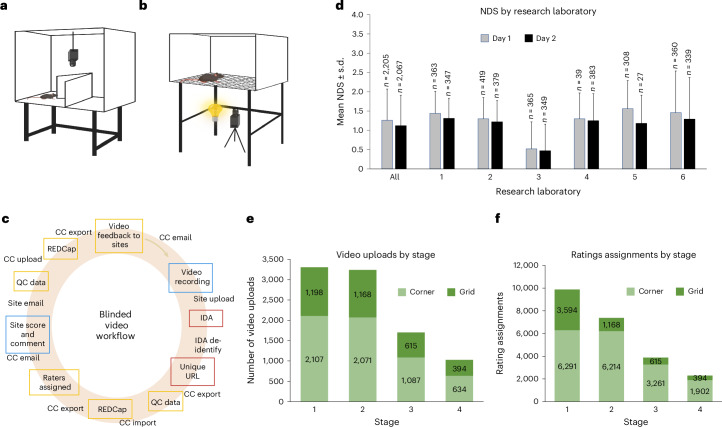
Behavioral testing workflow and feasibility. **a**, Sketch of the corner test setup with the camera mounted above the apparatus. **b**, Sketch of the grid-walk test setup with the camera and light source below the grid. **c**, Workflow of the behavior test: videos were recorded, uploaded and then sent out for blinded scoring. Multiple QC steps throughout the workflow assured data integrity. **d**, NDS of animals at day 1 and day 2 post-MCAo for each research laboratory and combined including all five animal models. Data represented as mean ± s.d. **e**, Numbers of corner and grid behavior test videos uploaded to IDA for each stage. **f**, Numbers of corner and grid behavioral test assignments completed by research laboratories for each stage. None of the research laboratories rated their own videos. Panel **a** reproduced with permission from ref. ^22^, AAAS.

The corner test was selected as the primary outcome assessment and used for the sample size/power calculation of the multistage statistical design^32,33^. The corner test is simple to perform without expensive or complex equipment (Fig. 4a). The grid-walk test (Fig. 4b) and hanging wire test were selected as secondary outcomes, but the hanging wire was eliminated after stage 1 for being redundant^34^. To ensure fully blinded, objective ratings of the behavioral assessments, anonymized digital video recordings of each evaluation were assigned to blinded raters at research laboratories other than the one that generated the recording (Fig. 4c). NDS^35^ indicated mild-to-moderate stroke severity across the entire study and were balanced across research laboratories (Fig. 4d), intervention and sex. Because interventions were dropped between stages, total uploads of recorded videos decreased over the four stages (Fig. 4e), as did the number of ratings generated from those uploaded videos (Fig. 4f).

The corner test proved feasible and baseline values were remarkably similar across the five different animal models^20,21^. Across all animal models and treatments, MCAo provoked substantial behavioral deficits at 7 days and 30 days^22^. While there was some heterogeneity across research laboratories, overall, the corner test indicated similar insult severity^21^.

**Extended Data Fig. 1:**
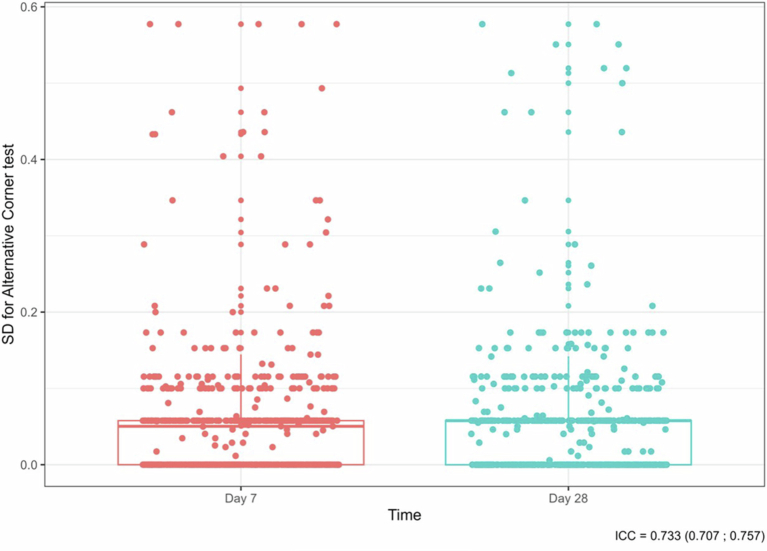
Variation among raters for the Day 7 and Day 28 corner test video. Each dot represents the standard deviation of 3 reviewer scores for one video taped corner test. Day 7 n = 617 and Day 28 n = 563. A box-and-whisker plot displays the median (central line inside the box), and the 25^th^ percentile or first quartile (lower edge of the box), the 75^th^ percentile or third quartile (upper edge of the box), and the whiskers, which extend to 1.5 times the interquartile range (IQR) from the quartiles. The ICC is shown with the 95% confidence range.

Each recorded corner test was randomly assigned to three certified raters. Recordings contained no identifying labels as to intervention or originating research laboratory. After stage 1, the intraclass correlation coefficient (ICC) was calculated to assess concordance among the three raters. Concordance among three human raters was good, but insufficient to reduce the number of raters (ICC 0.732; 95% confidence level 0.71 – 0.76), adjusting for time and intervention (Extended Data Fig. 1).

The grid-walk recordings and assessments were rated similarly to the corner test. However, at the end of stage 1, concordance among the three human raters was sufficient to reduce the number of raters to one in subsequent stages. Again, feasibility and completion rates were similar to the corner test results.

### Image analysis pipeline

**Fig. 5:**
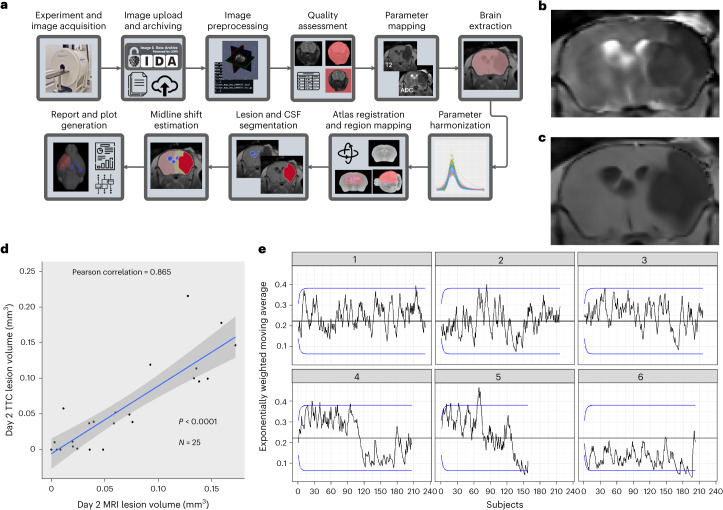
Lesion volume estimation using an automated image analysis pipeline. **a**, MR images of each subject were analyzed via an imaging pipeline. **b**,**c**, Imaging sequences included a T2-weighted scan (**b**) and an ADC-weighted image (**c**). **d**, In a pilot study, the fully automated analysis was validated using TTC-stained sections. Forty-eight hours after a 60-min MCAo, 25 young healthy mice were imaged, immediately euthanized, and their brains were sectioned into 2-mm-thick slices and stained with TTC. **e**, To assist the research laboratories with their protocol adherence over the course of the study, a control limit chart of day 2 lesion volume was sent on a regular basis. Control limits were defined as two s.d. around the study-wide mean lesion volume. CSF, cerebrospinal fluid. Panels **a**–**c** reproduced with permission from ref. ^36,IEEE^.

MRI of each subject was attempted 2 and 30 days after the stroke, and the anonymized data were analyzed via an imaging pipeline (Fig. 5a). Imaging sequences included a T2-weighted scan (Fig. 5b) and an apparent diffusion coefficient (ADC)-weighted image (Fig. 5c). In a pilot study^36^, the fully automated analysis was validated using 2,3,5-triphenyl tetrazolium chloride (TTC)^37^-stained sections (Fig. 5d). The resulting correlation was excellent (*r* = 0.86, *P* < 0.0001, *n* = 25). To assist the research laboratories with their protocol adherence over the course of the study, a control limit chart of the day 2 lesion volume (Fig. 5e) was sent on a regular basis^38^. Control limits were defined as two standard deviations (s.d.) around the study-wide mean lesion volume.

### Data quality assurance

**Fig. 6:**
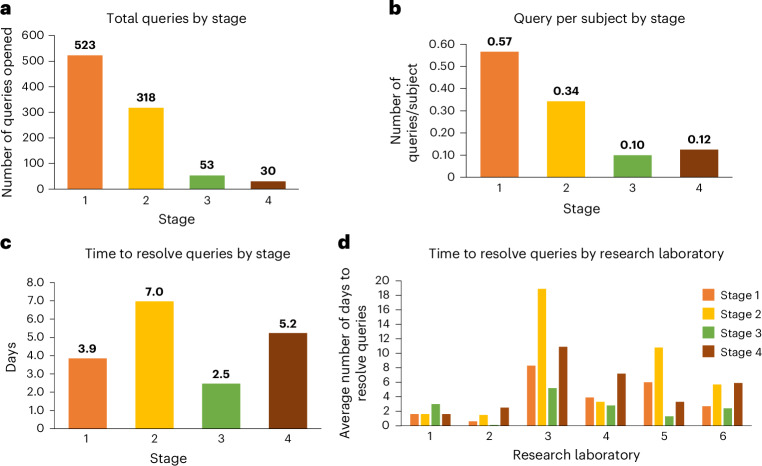
Queries. **a**, Total queries created in REDCap across the four stages of the trial, showing improved data entry. **b**, To account for the decreased number of subjects and interventions in each stage, the number of queries per subject was calculated; improvement persisted, with a slight increase observed in stage 4. **c**, Research laboratories took a varied number of days at each stage to resolve queries; however, the response time was still low. **d**, The chart shows the varied time that each research laboratory took to resolve open queries over the four stages of the trial.

Data monitoring at the CC ran continuously throughout all four stages of the study. The CC investigators checked key data elements using a risk-based monitoring approach drawn from clinical trial design^39,40^. Data discrepancies were resolved by issuing a data query to the relevant research laboratory, which then drew attention to certain fields to the data entry personnel. As a result, the total number of data queries dropped considerably over the four stages (Fig. 6a). Because the stages differed in the number of interventions and, therefore, the total number of subjects enrolled declined over the stages, the number of queries adjusted for the total enrollment in each stage was also compared (Fig. 6b), which again confirmed a definite improvement over time, suggesting the existence of a learning curve in this multisite preclinical experiment. One indicator of the commitment of the research laboratory is the time used to address and reply to the data queries. The research laboratories reduced their time to query resolution over the four stages (Fig. 6c), but there was notable variation among them (Fig. 6d). In future applications, network managers should be aware of the variable commitment to data quality across research laboratories and overtime, and plan quality control (QC) activities accordingly.

## Discussion

The successful design and execution of SPAN was founded on experience gained from two prior multisite network-based studies^41,42^. We created and adopted network-wide SOPs, including one common stroke model (filament MCAo) in five animal models that all six research laboratories embraced. The MAMS statistical procedure allowed SPAN to rigorously identify interventions that fell below the futility boundary, sequentially eliminate them and to continue testing those that had not exceeded the efficacy boundary. After four stages, one intervention, (the free-radical scavenger uric acid)^20,43^ exceeded the efficacy boundary. The feasibility of the approach is further supported by the observation that SPAN began subject enrollment during a global pandemic with intermittent, mandatory lockdown of laboratory staff. SPAN took advantage of digital, internet-based video conferencing, video training and centralized certification to maintain progress during coronavirus disease 2019 pandemic lockdown periods.

Traditionally, substantial organizational barriers impede the implementation of a multilaboratory collaboration. The SPAN investigators addressed several of these barriers. SPAN drafted a template application for the research laboratories to submit to their Institutional Animal Care and Use Committees after assuring compliance with the NIH and other national guidelines^44^. Research laboratory contracting was handled via an NIH grant mechanism (RFA NS-18-34 and NS-18-033) but could have been challenging in a privately funded effort. Future planners should allow time for contract negotiations. A plethora of decisions was made involving every aspect of the study protocol. Decision-making required finding consensus after careful and thorough literature review of options and prior experience, a collaborative approach guided by the SC and a commitment from investigators to reach agreements on key decisions expeditiously. Minor operational decisions (for example, the color of the labels or brand of the vial boxes) were made by CC investigators for efficiency, and detailed decision logs were maintained by the CC.

Enrollment proceeded toward a prespecified sample size, based on the agreement that each research laboratory could enroll eight subjects per week. This enrollment rate proved feasible at all six research laboratories over the 2.5 years of enrollment. Furthermore, careful coordination of behavioral assessments and scheduling access to the MR scanners was necessary. To enhance feasibility and timely throughput, the SPAN CC created laboratory-specific ordering and surgery timelines that were distributed to all research laboratories prior to each of the four stages (Supplementary Table 3). Figures 1–3 provide descriptive data about many aspects of the trial. Collectively, these data paint a picture of the expected workload for future planners to set up their own preclinical assessment network.

The SPAN investigators created operational methods to implement several desirable elements deemed essential to rigor in preclinical trial design^14–16,45^. All drug interventions were packaged in similar-looking, labeled vials (Fig. 3a and Supplementary Fig. 2) to conceal the treatment assignment from the investigator who induced the disease model. Central randomization and 100% assignment of subjects avoided attrition bias and selection bias (Fig. 2a). By distributing anonymized digital outcome data (behavior videos or MR images), group blinding was preserved through final data analysis (Figs. 1d and 4c). Given that rigor and scrupulous experimental design might enhance the likelihood of future success in clinical trials, the approach presented here provides future investigators with a feasible and practical approach.

From the outset, the methods created for SPAN and presented here were intended to serve more than one project. Principles of rigor and scientific excellence, for example, concealment, blinding, randomization and statistical power, transcend any specific intervention focus and generally apply to other disease areas. The data presented confirm that the same methods performed well in different stages of the investigation, across five different animal models: young adult or aging C57BL/6J mice, obesity-induced hyperglycemia in C57BL/6J mice, young adult Sprague-Dawley rats and spontaneously hypertensive rats. Although the same approach to inducing stroke—the filament MCAo—was used across the study in all models, success in all five animal models suggests that the procedures and operational methods could work in other disease models. The SOPs were also written to allow adaptation to any other disease areas. Although we believe our methods can be applied to other disease areas where investigators seek to assess putative disease-modifying intervention in animal models, our network has so far used only one method for inducing stroke. Generalizability is suggested, but not a given. This study focused the assessment on only a few outcomes; other outcomes could perform differently, perhaps with varied concordance.

Notice must be taken of results in the aging mouse model. It has been suggested that putative stroke interventions must be tested in the aging model with comorbid conditions. However, the mortality of MCAo in aging mice was over 50%. During this investigation, SPAN investigators attempted multiple maneuvers that have been described by others to promote survival in these mice: scrupulous temperature control, careful anesthesia, fluid resuscitation, limited handling and careful genital hygiene in aging males. Unfortunately, over the course of stages 2 and 3, which together included over 340 aging mice, we could not improve survival. Although stroke in aged patients has higher mortality than in younger patients, the mortality is nowhere near 50%^46^. Furthermore, from a cost and efficiency perspective, and even perhaps reproducibility, MCAo in aging mice may not be a viable future option for assessing putative interventions, although this question deserves further study.

The methods presented here for establishing and maintaining a preclinical disease intervention network are feasible, practical and generalizable. In addition, the specific operational methods should be straightforward to implement in other disease areas.

## Methods

### Trial setup

SPAN investigators began meeting in 2019 to select and design all aspects of the SPAN network, including structure, communication, animal models, outcomes and protocols. The SPAN CC drafted clearly defined SOPs for all activities, which were edited and approved by the SC. An in-person kick-off meeting was held in Los Angeles, CA, on 9 and 10 September 2019, at which time protocol decisions were made. The CC also created and managed a separate Imaging Committee to design and approve the MRI protocol used in SPAN and will be available by contacting spancc@usc.edu.

### Network governance

To expedite decision-making and to oversee protocol development, the governing body of the network was a SC, convened by the CC in conjunction with the National Institute of Neurological Disorders and Stroke (NINDS) (Fig. 1b). SC membership included the CC PI, the PI of each research laboratory, and NIH Program scientists. In addition, an independent External Advisory Board (EAB), appointed by and reporting to NINDS, was chartered to include basic, translational and clinician scientists with expertise in cerebroprotection, representatives from the pharmaceutical and biotech industry, and experts in regulatory affairs, statistics and clinical trial design.

### Network capability assessment

Upon notification by NINDS of the six research laboratories selected for SPAN, the CC conducted in-person visits to assess the infrastructure available at each research laboratory (Fig. 1a). Surgeon experience, resources available at the research laboratory, MRI capability and several other elements were summarized. This compiled review of capabilities allowed the CC to create experimental protocols that would be feasible across all research laboratories. Communication systems were built, including a group email address at each research laboratory so that all team members could be contacted simultaneously. The CC established a hierarchy and organization for the flow of information to and from research laboratories and to the imaging repository (Fig. 1d).

### Optimized communication

The SPAN CC team was in daily contact with staff at each research laboratory via email and phone. SC meetings were held monthly, and stage-specific meetings were held weekly. Initially, the SC meetings were limited to the research laboratory PIs but were expanded later to include all hands-on investigators to improve communication and understanding of the protocol and SOPs at the research laboratories. The SPAN CC sent a weekly Enrollment Report to the research laboratories, the NINDS and the EAB. The CC team visited each research laboratory twice in person and once virtually during the project. These visits allowed the CC to inspect the surgical and behavior-recording areas, audit data, meet staff and disseminate best practices. The SPAN website (www.spannetwork.org) was used as a repository for distributing the SOPs and other needed information to all research laboratories. Within the website, a public-facing webpage contains general information about the project and its members. A private-access page allowed the CC to post template documents and SOPs. The website included a chat forum for investigators to share ideas and experiences.

### Decision-making

A modified version of the Delphi method was used to resolve conflicts and reach consensus. This approach began with monthly Network meetings, which later shifted to weekly sessions to facilitate the inclusion of comorbid models and protocol development. Surveys were sent to PIs before each meeting to stimulate discussions and gather initial feedback. During the meetings, participants received summaries of the responses, and discussions focused on areas of agreement and disagreement, encouraging further deliberation. After thorough discussions, the Network voted on decisions, with meetings following structured agendas that outlined the topics for decision-making. The final decision was determined by a majority vote. In addition, the EAB was convened to provide expert feedback, offering an objective perspective on the deliberations. The final decisions reflected the collective input of the group, emphasizing areas of consensus and converging opinions.

### Conflict resolution

The SPAN CC sought to facilitate open, transparent communication and encouraged robust discussion on all topics. Consensus was achieved gradually and thoughtfully with multiple rounds of review. The NINDS Scientific Officers retained final authority to settle disputes if needed, but this never became necessary during the study. The CC regulated the decision-making deadlines to meet study timelines and coordinated a timely discussion through forums and emails with the SC. A pilot stage, consisting of ten subjects per research laboratory, was helpful in troubleshooting the workflow of data collection, surgical procedures behavior testing and video upload.

### Interventions, concealment and blinding

Through rigorous peer review, six interventions were selected for study in this project^20^. One mechanical procedure, called remote ischemic conditioning, needed its own control group, making it impossible to conceal group assignments. The remaining five compounds (uric acid, tocilizumab, veliparib, fingolimod and fasudil) were formulated as either IV or IP infusions. The CC met with biopharmaceutical companies and arranged for either purchased or donated drug interventions (Supplementary Note 1). Detailed information was collected from the investigators or manufacturers about preparation, valid excipients, aliquoting and storage. The CC tried to obtain stability data on each compound. Where possible, drug interventions were resupplied with expiration dates based on this stability data. Where such stability information was not available, the CC conducted stability trials in-house using high-performance liquid chromatography (uric acid) or shipped test vials to research laboratories for them to perform bioassays (uric acid, fasudil and fingolimod). In addition, as a further demonstration of stability, repeat bioactivity assays were performed on select drug interventions at the end of stage 1 with unused vials at the research laboratory. These unused vials were tested near the end of their expiration dates, and all bioactivity was preserved.

To facilitate blinding, the CC chose to administer two of the drug interventions IP (fasudil and fingolimod) and three of the drug interventions IV (veliparib, tocilizumab, uric acid) with matching 0.9% weight/volume (w/v) saline placebo controls. Dosing (sex and species) was determined by the research laboratory that proposed the intervention. The IV drug interventions were shipped in liquid form for a single 8 µL/g body weight infusion to be administered over 20 min starting 5 min before reperfusion^47^. The IP drug interventions were shipped in a vial that contained enough volume for each animal it was assigned to. The first IP injection was administered 5 min before reperfusion and then twice daily for five more doses (six doses in total). One of the IP drug interventions had a shorter shelf life once suspended, so it was decided that all IP drug interventions would be lyophilized and resuspended immediately before its first use. Because of the larger volume required for six injections in 0.9% w/v saline, IP drug interventions were lyophilized in 5% w/v saline to minimize volume and prevent boil-over during lyophilization. For each randomized subject, an email prescription was generated that indicated the exact volume of sterile water to add to the coded vial to result in a final concentration of 0.9% w/v saline, and the correct concentration of resuspended drug interventions. For each stage, the CC located and purchased glass vials (Supplementary Table 2) that were large enough to hold required quantities of drug interventions after estimating the expected dose ranges given the ages and weights of the mice and rats to be included (Supplementary Fig. 1a). At the CC, all vials were sealed with nonreactive rubber stoppers and secured with a crimped flip cap to maintain sterility of the septum until use (Supplementary Note 1). Placebo vials were prepared identically by loading 0.9% w/v saline or lyophilizing 5% w/v saline into matching vials. Multivial boxes (Fig. 3b) for each research laboratory were loaded at the CC with the vials needed for one randomization block. Boxes were then labeled and shipped in qualified 2-day summer shipping containers (ThermoSafe E3R2S, E6RR2S, E12R2S) with a temperature tag (ShockWatch WarmMark: WM 8/46). Research laboratories could read only the CC-applied labels and could not identify which intervention the vial contained.

### Data collection and organization

Using REDCap^48,49^ (research electronic data capture), a project was designed for data entry for both the end users and the CC. Several repeatable forms were designed to capture data over four stages of SPAN. Data access groups were established so that each research laboratory received access to their own records only. The REDCap Alerts & Notifications application was used to trigger email signals to notify the CC when subjects were ready for randomization and to notify the research laboratory when randomization was completed. Custom reports were created in REDCap to track intervention accuracy, scheduled versus actual surgery dates, and incoming rater scores for invalid or missing fields, among many others. These reports facilitated QC and management.

Using Excel and the Visual Basic Macro Language, the Master intervention spreadsheet (MISS) was designed to track inventory and assign vials during the manual randomization process (Supplementary Table 4). Using the MISS, CC staff tracked all inventory upon arrival at the CC, during shipment to the research laboratories, and as it was administered at the laboratories. The MISS also calculated the dose to administer and provided an easy way to monitor for the shelf-life expiration of each vial. The MISS used relational logic to cross-check fields and had preprogrammed alerts (Supplementary Note 3 and Supplementary Table 5).

### Animal models used

To increase the translational relevance of the study, we included disease models that incorporated some of the key risk factors for stroke, including age, hypertension and hyperglycemia. After a successful first stage using young, disease-free subjects, comorbidities were included in stages 2 and 3. The comorbid models used were: aging mice, diet-induced obesity in mice and spontaneously hypertensive rats. Aging mice, 15–17 months at surgery, were obtained from the NIA (Aged Rodent Colonies, NIA (nih.gov)). To induce obesity, C57BL/6J mice were fed a high-fat diet (EnvigoTeklad; TD.06414) for 12 weeks and were 15–18 weeks old at surgery^50–52^. Spontaneously hypertensive rats (SHR/NCrl) were obtained from Charles River Laboratories^53^ and were 15–17 weeks old at surgery. To ensure even distribution of experience across all research laboratories and to avoid either research laboratory practice effects or seasonal effects, we instituted several measures: the CC randomized the models assigned to the research laboratories in each stage; the randomization process (drawing of lots) was recorded using a video camera and archived for later review if needed. During stages 2 and 3, each research laboratory was assigned to perform two of the models; no more than two research laboratories could perform a particular model simultaneously; research laboratories had to alternate between their assigned two models at least twice per stage to prevent an experience effect. Because there was only one animal model in stage 4, no rotations were necessary. By the end of the study, all models were performed in at least four different research laboratories. All these scheduling requirements ensured that the study was free of practice effects, research laboratory preference/experience with one or another model and seasonal effects.

### Surgical procedures

All animal protocols were approved by the Institutional Animal Care and Use Committees: Site 1 AUP#2020-1026, Site 2 0032291 then 0032291-05, Site 3 MO19M154 then MO22M30 and RA21M130, Site 4 2020N000058 and 2021N000089, Site 5 AWC-20-0063 and AWC-20-0079 and Site 6 2019-20282.

For this trial, the filament MCAo^54^ was implemented at all research laboratories for several reasons. First, all research laboratories had prior experience performing a version of it, which reduced training and start-up time. Second, the filament model can be easily accomplished in both mice and rats. Third, with the advent of thrombectomy in clinical practice^55^, in which an intravascular clot-retrieval catheter is used to recanalize an occluded artery, it is now possible to know precisely when reperfusion begins and then immediately start intervention. We used the filament MCAo model to represent this clinical scenario of known recanalization with immediate initiation of the intervention. The SC drafted and agreed upon experimental protocols for each of the four stages, including standardization of ischemia and anesthesia. All SPAN experimental protocols are available by contacting spancc@usc.edu

To ensure surgical reproducibility, all surgeons were certified before starting work. They performed the MCAo surgeries until they could produce ten surgeries with measurable lesions, as demarcated with TTC. Once the CC received the TTC images and morphometry, these were reviewed, and if satisfactory, the surgeon was approved to begin. This process allowed new surgeons to join the network at any time.

### Process control

Throughout the trial, the CC desired to monitor quality across all research laboratories. To monitor stroke volume as a key indicator of research laboratories quality, we used a control limit chart^56^ approach using the exponentially weighted moving average that weighted more recent data more heavily^38^:

$${M}_{i}={\lambda M}_{{\rm{o}}}+\left(1-\lambda \right){M}_{i-1},$$

where ${M}_{{\rm{o}}}$ is the average of volume lesion from the surgeon certification at stage 1 or volume fraction from stage 1 for the remaining stages; ${M}_{i}$ is the current moving average; and ${M}_{i-1}$ is the previous moving average. The control limits were established based on the network-wide mean value ${M}_{{\rm{o}}}$ ± *L* × ${V}_{i}$, with ${V}_{i}$ given by

$${V}_{i}^{2}={\sigma }_{0}^{2}\left(1-{\left(1-\lambda \right)}^{2i}\right)\lambda /(2-\lambda ),$$

where ${\sigma }_{0}^{2}$ is the variance of volume lesion from the surgeon certification at stage 1 and volume fraction from stage 1 for the remaining stages. Based on simulation studies, CC established $\lambda =0.15$ and *L* = 3. Charts were updated every 2 weeks, allowing research laboratories to monitor their performance relative to the control limits. At a few points in the trial, a research laboratory was briefly out of control. At these points, the CC informed the research laboratory and met with the surgical team to investigate potential explanations—such as changes in equipment—for the out-of-control variations. In all cases, the research laboratories corrected procedures and returned the metric to within control limits (Fig. 5e).

### Standardized behavioral assessment

To assure quality behavioral assessments, the CC arranged for online webinars during the global pandemic lockdown to illustrate proper technique. After each interactive webinar, research laboratories were given sample test videos to rate. After correctly scoring these test videos, an investigator was then considered certified to rate behavior in the study. Test videos included intentional violations of the recording SOP, and the viewers were expected to detect and label >90% of all these protocol violations. Raters who failed to correctly score the test videos, including failure to comment on protocol violations, were asked to rewatch the recorded training webinar and then rate a new set of test videos. This approach not only enabled rigorous initial training and certification of raters but also allowed new raters to join the trial at any later time.

### Standardized video recording

The corner test (Fig. 4a) and grid-walk test (Fig. 4b) were both recorded at the research laboratories to be scored later by blinded, certified reviewers at other laboratories. Each research laboratory used a digital camera (GoPro Hero 7, 8 or 11), set to the lowest possible resolution (preferred 720 pixels) and frame rate (preferably 30 frames per second) to reduce file size and time needed to upload and download the videos for scoring. Standardized lighting was required to allow an optimum view of the behaving subject. The resulting, recorded digital video files were uploaded to the IDA, a repository for the long-term preservation and sharing of biomedical research data^27^.

### Standardized video scoring/rating

Digital recordings of the corner and grid-walk tests were assigned at random by the CC to three trained and certified raters at research laboratories other than the one that recorded the video. Each week, raters received behavior scoresheets with lists of anonymized URL addresses and empty fields for data entry. Each rater scored the assigned video and entered the results into the scoresheet. Investigators were given 1 week to return completed scoresheets. After running quality checks on the returned data, the CC imported these results into the REDCap database. Raters did not know the identity of the research laboratory, sex, behavior time point or intervention given to the subject in the video they rated.

### Randomization

Centralized randomization assures that subjects are allocated to intervention groups without bias or baseline differences. Stratification during randomization ensures balanced numbers in key variables that could influence outcome, such as sex and research laboratory; other stratification variables may apply in other disease-specific implementations. These needs required the design of a manual process that contained three essential tools: REDCap database, a MISS and a set of randomization tables generated for each research laboratory. These tools are described in detail to facilitate use in other preclinical assessment networks.

Subjects began the centralized randomization process when the participating research investigator assigned the subject to a surgery date using an ITT form in REDCap. Stroke surgeries for males and females were to be performed concurrently and evenly distributed during each surgery day. Upon notification by the ITT form, the CC consulted the appropriate randomization table and assigned the subject to the next available row. The intervention assignment was entered into REDCap and the MISS (see Supplementary Note 3 for a more detailed description of the randomization process). After QC steps to assure correct treatment assignment, a randomization email was sent to the research laboratory containing all information needed to treat: subject ID, assigned vial number, administration volume, route of administration and the volume of sterile water to add to resuspend if IP and intervention schedule. After stroke surgery was performed, and intervention administered, correct administration was confirmed post hoc.

### Randomization tables

The randomization tables were created by the statistical team using the prespecified analysis plan, the stratification variables and knowledge of the number of research laboratories and number of interventions. The tables and the MISS were devised in close collaboration. Rather than use a permuted block size, the randomization block sizes were unique to each stage and calculated from multiples of the weekly subject enrollment at each research laboratory, the total sample size, the sex, the model and the number of interventions being studied. Such variation in randomization block sizes allowed for equal distribution of interventions across sexes and models, ensured adequate rotation of models, improved timing efficiency for resupplying interventions to research laboratories, and reduced loss due to expired vials. Laboratory investigators were unaware of this approach to randomization block sizes.

### Avoiding bias from attrition, selection and performance

Every animal purchased for SPAN was tracked through the entire protocol (Fig. 2b). Upon arrival at the research laboratory, a barcoded ear tag (RapID) was affixed (Fig. 2a and Supplementary Fig. 1c). Barcodes were read with a handheld scanner paired to a Bluetooth-compatible device to ensure accurate ear tag entry. The research laboratory documented the ear tag color in the enrollment form. The ear tag color was verified during subsequent study procedures, such as during behavior video review and scoring. If a discrepant animal ID or tag color was noted, the animal record was flagged and investigated. Once enrolled, 100% of subjects were accounted for through the end of the study. To prevent surgeons from assigning interventions to individuals in a biased manner, we used group concealment^45^, described above. To assure unbiased assessment, the investigators performed all behavior ratings and image analysis blinded to any knowledge of intervention group assignment^12,14,57^. The methods are described above.

### MRI methods

A protocol for MRI included quantitative imaging sequences for estimating the ADC and T2 relaxation rate. Data were acquired from Bruker scanners with field strengths across research laboratories, including 7 T, 9.4 T or 11 T. A multi-echo T2 sequence was used to estimate the T2 rate using relaxometry, with echo times rating from 10 to 100 ms. A diffusion-weighted imaging sequence was used to estimate ADC, with *b*-values 0, 500 and 1,000 s/mm^2^. After data acquisition, image files were uploaded to the IDA of the LONI for long-term storage and subsequent analysis. Using the Quantitative Imaging Toolkit^58^, we created an automated image analysis pipeline (code posted at 10.48550/arXiv.2203.05714) for generating quantitative reports and visualizations of individual animals, summarized^58^ in Fig. 5a. We segmented the brain using a deep learning neural network approach with a U-net architecture^59^, and we segmented lesions, cerebrospinal fluid and normal-appearing brain tissue using a combination of thresholding and mathematical morphology. We estimated midline shift by isolating cerebrospinal fluid closest to the midline, computing the centroid and measuring the relative distances to the boundaries of the brain. To visualize our data in a common reference frame, we aligned our data to a group-averaged scan using rigid registration with Advanced Normalization Tools^60^. We measured the volume of segmentation brain areas and lesion, aggregated the results in tabular form and generated mosaic plots for later qualitative assessment.

### Data management

Data inconsistencies across forms in the database, or range-check errors, resulted in a data query from the CC to the research laboratory. Data queries (Fig. 6a,b) were initiated by the CC using the REDCap database. Research laboratory technicians and PIs were instructed to check REDCap data queries frequently. If the laboratory did not resolve the query initiated in REDCap within 48 h, the CC alerted the technician to the query by email. If queries went unresolved for longer, the CC would request that the research laboratory PI follow up with their technician to resolve the query (Fig. 6c,d). Daily, the CC ran quality checks in REDCap to track study progress (surgery completion, dropouts, intervention administration and end of study) at each research laboratory and to reconcile video and MR image uploads into IDA. When inconsistencies in video and MRI uploads were identified, the CC contacted investigators at the research laboratory to upload the missing video or image (Supplementary Note 2).

### Statistical analysis

Interventions were assessed using a MAMS design based on the generalized Dunnett’s test^24,25^. The MAMS design provides large gains in efficiency over separate randomized trials of each treatment as it allows a shared control group. Futility (lower) and efficacy (upper) boundaries were calculated in advance. At each interim analysis, a Dunnett test statistic for the primary endpoint comparing each arm with the control arm as reference was calculated^61^. If the test statistic value fell below the futility boundary, the intervention was to be declared as nonpromising, and enrollment into that arm was to stop. If the test statistic value fell above the efficacy boundary, the intervention was to be declared effective, and enrollment into that arm could be stopped. Otherwise, the intervention was declared promising, and enrollment into that arm continued into the next stage.

### Power and sample size considerations

Two parallel multistage trials were performed with the corner test index as the primary endpoint after transformation. The power analysis used this variable to derive sample sizes: (A) the first trial included five drug intervention arms and one control arm, split equally between IP control and IV control under the assumption that there would be no difference between the two control treatments for the primary endpoint; (B) the second trial had one mechanical intervention arm and one control arm. We implemented the MAMS design with four stages using triangular futility and efficacy boundaries^23,25^. Three interim analyses were planned after 25%, 50% and 75% recruitment with an equal allocation among arms. Based on a published study of the corner test in aged subjects, we estimated a mean of 0.55 and a s.d. of 0.262 for the control arm^32^. We then increased the assumed s.d. to 1.048 as a conservative scenario to account for predicted excessive variability among the research laboratories. Assuming that the transformed corner test index in different animal models follows independent normal (*μ*, *σ*^2^) distributions with known variance, we defined an interesting effect size (*δ*) as a decrease on the corner test index of at least 50% to declare an intervention as effective, and a non-interesting effect size (*δ*_0_) as a decrease of at most 6% to declare an intervention as ineffective. We chose 50% for the efficacy boundary based on a meta-analysis of prior preclinical trials in which the mean reported effect size was 50%^62^. We chose 6% for the futility boundary as this value was the effect size used to power several large, phase 3 trials of interventions for acute ischemic stroke^63,64^. We tested a family of null hypotheses (H_0_), each stating that the intervention arm mean is greater than or equal to the control arm mean for the 30-day transformed corner test index. The family-wise error rate was defined as the probability of falsely rejecting one or more null hypotheses within this family at any trial stage with a fixed value of 5%. The power of 90% was calculated under the least favorable configuration, which is defined as the probability of rejecting only one null hypothesis in any stage^65^. We computed the minimum, maximum and expected sample sizes under the null and least favorable configuration hypothesis based on triangular boundaries^66–68^. Total planned sample size was then adjusted assuming 10% animal death over 30 days for the entire experiment including all five animal models.

## Online content

Any methods, additional references, Nature Portfolio reporting summaries, source data, extended data, supplementary information, acknowledgements, peer review information; details of author contributions and competing interests; and statements of data and code availability are available at 10.1038/s41684-026-01683-z.

## Supplementary information

Supplementary Information Supplementary Note 1: Intervention concealment including comments regarding color, volumes/dosing, storage, preparation, packaging, bottling, labeling and shipping. Includes Supplementary Figs. 1–3. Supplementary Table 1: Maximum tolerable doses for rat and mouse species. Supplementary Table 2: Vial product numbers used for mouse/rat species. Supplementary Fig. 1a–c: Picture of unlabeled SPAN vials used in different stages (a), 3D-printed 30-mL vial tray (b) and RapID tags/applicator (c). Supplementary Fig. 2: Picture of vial boxes J and K for two research laboratories. Supplementary Fig. 3: SPAN Stage 3.0 CC shipping schedule. Supplementary Note 2: Behavior testing and rating concealment including video preparation and blinding, data transfer security and interconnectivity, rater/recorder certification, video assignment and video feedback to research laboratories. Supplementary Note 3: Research laboratory organization and best practices include randomization and QC. Includes Supplementary Fig. 4. Supplementary Fig. 4: CC workflow for randomization and verification

Supplementary Tables 3–5 Supplementary Table 3: Research laboratory sample scheduling timeline. Supplementary Table 4: Sample-blinded MISS used for tracking inventory, vial assignments, expiration and dose. Supplementary Table 5: Formulas and conditions used to create the MISS Excel file.

## Data Availability

The data (including SOPs, experimental protocols, MRI pipeline raw data and code) that support the findings of this study are available from the corresponding author upon request.
