# Author Correction: Methods for randomized, blinded, controlled evaluation of putative disease interventions in multilaboratory, preclinical assessment networks

**DOI:** 10.1038/s41684-026-01709-6

**Published:** 2026-03-05

**Authors:** Jessica Lamb, Karisma Nagarkatti, Marcio A. Diniz, Ryan Cabeen, Monica Estrada, Karen L. Crawford, Andre Rogatko, Sungjin Kim, Cenk Ayata, David C. Hess, Mohammad Badruzzaman Khan, Rakesh B. Patel, Mariia Kumskova, Enrique C. Leira, Anil K. Chauhan, SPAN Consortium, Kazi Akhter, Ken Arai, Ali Arbab, Jaroslaw Aronowski, Brooklyn Avery, Hannah Beatty, Adnan Bibic, Frank Blixt, Ligia Boisserand, Marian Cabrera-Ayala, Suyi Cao, Anjali Chauhan, Valina Dawson, Kris Dhandapani, Nirav Dhanesha, Sebastian Diaz, Taylan Erodogan, Andrew Goh, Ali Herman, Shuning Huang, Fahmeed Hyder, Takahiko Imai, Emma Imakavar, Xuyan Jin, Conor Johnson, Pradip Kamat, Senthilkumar Karuppagounder, Raymond Koehler, Ewa Kulikowicz, Javier Labastida, Steven Lannon, Siyue Li, Eng Lo, Joe Mandeville, Michael Maniskas, Louise McCullough, Andreia Morais, Diego Motales-Scheihing, Steward Niefert, Mohammad Nisar, Lidiya Obertas, Tao Qin, Basavaraju G. Sanganahalli, Lauren Sansing, Yanrong Shi, Shahneela Siddiqui, Cameron Smith, Guanghua Sun, Brijesh Sutariya, W. Taylor Kimberly, Dan Thedens, Shun-Mug Ting, Klaus van Leyen, Peter Van Zijl, Sofia Velazquez, Jun Wang, Nicholas Wilder, Kristofer Wood, Jiadi Xu, Lili Yu, Steve Zeiler, Patrick Lyden

**Affiliations:** 1 Department of Physiology and Neuroscience of the Zilkha Neurogenetic Institute of the Keck School of Medicine, Los Angeles, CA USA; 2 Biostatistics and Bioinformatics Research Center, Samuel Oschin Comprehensive Cancer Center, Cedars-Sinai Medical Center, Los Angeles, CA USA; 3 USC Stevens Neuroimaging and Informatics Institute, Los Angeles, CA USA; 4 Neurovascular Research Unit, Department of Neurology, Harvard Medical School, Massachusetts General Hospital, Boston, MA USA; 5 Department of Radiology, Harvard Medical School, Massachusetts General Hospital, Charlestown, MA USA; 6 Department of Neurology, Medical College of Georgia, Augusta University, Augusta, GA USA; 7 Department of Internal Medicine, Division of Hematology, Oncology and Blood and Marrow Transplantation, Carver College of Medicine, University of Iowa, Iowa City, IA USA; 8 Department of Neurology, Carver College of Medicine, University of Iowa, Iowa City, IA USA; 9 Department of Neurosurgery, Carver College of Medicine, University of Iowa, Iowa City, IA USA; 10 Department of Epidemiology, College of Public Health, University of Iowa, Iowa City, IA USA; 11 Department of Neurology of the Keck School of Medicine, Los Angeles, CA USA; 12 Department of Radiology, Johns Hopkins University, Baltimore, MD USA; 13 Department of Neurology, Johns Hopkins University, Baltimore, MD USA; 14 Biochemistry and Molecular Biology, Medical College of Georgia, Augusta University, Augusta, GA USA; 15 Department of Neurology, McGovern Medical School, University of Texas HSC, Houston, TX USA; 16 Department of Neurology, Yale University School of Medicine, New Haven, CT USA; 17 Department of Neurology, Institute of Cell Engineering, Johns Hopkins University, Baltimore, MD USA; 18 Neurosurgery, Medical College of Georgia, Augusta University, Augusta, GA USA; 19 Department of Immunobiology, Yale University School of Medicine, New Haven, CT USA; 20 Department of Biomedical Engineering, Yale University, New Haven, CT USA; 21 Department of Anesthesiology and Critical Care Medicine, Johns Hopkins University, Baltimore, MD USA; 22 Department of Immunology, MD Anderson Cancer Center, Houston, TX USA; 23 Department of Radiology and Biomedical Imaging, Yale University, New Haven, CT USA; 24 Center for Genomic Medicine and Division of Neurocritical Care, Department of Neurology, Massachusetts General Hospital, Boston, MA USA; 25 Radiology, College of Public Health, University of Iowa, Iowa City, IA USA; 26 Present Address: Department of Population Health Science and Policy, Icahn School of Medicine at Mount Sinai, New York, NY USA

**Keywords:** Research data, High-throughput screening, Research management

Correction to: *Lab Animal* 10.1038/s41684-026-01683-z, published online 18 February 2026.

In the version of this article initially published, the name listed for Basavaraju G. Sanganahalli appeared incorrectly (Basavaraju Sanganahilli Ganganna), and is now amended in the HTML and PDF versions of the article.

