# Supplementary material for: Methods for randomized, blinded, controlled evaluation of putative disease interventions in multilaboratory, preclinical assessment networks
Source: Lab Anim (NY). 2026 Feb 18;55(3):74–82. doi: 10.1038/s41684-026-01683-z (PMC12956586; doi:10.1038/s41684-026-01683-z)
Supplement: Supplementary file 1 — Supplementary Note 1: Intervention concealment including comments regarding color, volumes/dosing, storage, preparation, packaging, bottling, labeling and shipping. Includes Supplementary Figs. 1–3. Supplementary Table 1: Maximum tolerable doses for rat and mouse species. Supplementary Table 2: Vial product numbers used for mouse/rat species. Supplementary Fig. 1a–c: Picture of unlabeled SPAN vials used in different stages (a), 3D-printed 30-mL vial tray (b) and RapID tags/applicator (c). Supplementary Fig. 2: Picture of vial boxes J and K for two research laboratories. Supplementary Fig. 3: SPAN Stage 3.0 CC shipping schedule. Supplementary Note 2: Behavior testing and rating concealment including video preparation and blinding, data transfer security and interconnectivity, rater/recorder certification, video assignment and video feedback to research laboratories. Supplementary Note 3: Research laboratory organization and best practices include randomization and QC. Includes Supplementary Fig. 4. Supplementary Fig. 4: CC workflow for randomization and verification [file 41684_2026_1683_MOESM1_ESM.pdf]

lab animal

Supplementary information

https://doi.org/10.1038/s41684-026-01683-z

Methods for randomized, blinded, controlled evaluation of putative disease interventions in multilaboratory, preclinical assessment networks

In the format provided by the authors and unedited

<u>**Supplementary Materials: Table of Contents**</u>

- **Supplementary Note 1**: Intervention concealment including comments regarding color, volumes/dosing, storage, preparation, packaging, bottling, labeling, and shipping. Includes Supplementary Figure 1-3.
    - Supplementary Table 1: Maximum tolerable doses for rat and mouse species
    - Supplementary Table 2: Vial product numbers used for mouse/rat species
    - Supplementary Figure 1 (a-c): (a) Picture of unlabeled SPAN vials used in different stages, (b) 3-D printed 30mL vial tray, and (c) RapID tags/applicator
    - Supplementary Figure 2: Picture of vial boxes J and K for two research laboratories
    - Supplementary Figure 3: SPAN Stage 3.0 Coordinating Center shipping schedule.

- **Supplementary Note 2**: Behavior testing and rating concealment including video preparation and blinding, data transfer security and interconnectivity, rater/recorder certification, video assignment, and video feedback to research laboratories.
- **Supplementary Note 3**: Research laboratory organization and best practices include randomization and quality control. Includes Supplementary Figure 4.
    - Supplementary Figure 4: Coordinating Center workflow for randomization and verification
    - Supplementary Table 3: Research laboratory sample scheduling timeline
    - Supplementary Table 4: Sample blinded Master intervention spreadsheet (MISS) used for tracking inventory, vial assignments, expiration, and dose
    - Supplementary Table 5: Formulas and conditions used to create the Master intervention spreadsheet (MISS) Excel file.

Supplementary Note 1

Intervention Concealment

Concealment prevents selection bias when the investigator selects animals for the stroke procedure. SPAN interventions included five drugs and one mechanical intervention. Since it was impossible to conceal the mechanical intervention from its sham, this portion of SPAN was not concealed from the surgeon; all outcomes remained blinded. For each drug intervention, we determined the ideal route, dose, frequency, light sensitivity, shelf life, storage temperature, biohazards, and solubility when available; this information was needed to design effective concealment.

Color

To assure effective concealment, all drug interventions must appear the same color; fortunately, in SPAN, all drug interventions were clear in solution, but in other networks if one or more solutions were colored, the CC could add color to all vials. Amber vials could also be used to mask the solution color when on the shelf but once withdrawn into a syringe, the color could be revealed and unblind the investigator which would require additional steps to maintain blinding (Supplementary Fig. 1a)

Volumes and Dose

To maintain blinding, investigators should not be able to deduce the identity of the intervention based on the volume administered. To accommodate this, drug intervention concentrations were adjusted so that the research laboratory investigator administered a uniform volume. After considering the solubility of all five study interventions, the CC selected a volume of 8μl/gram (8ml/kg) body weight per dose for both rats and mice and for IV (slow bolus) and IP routes. So, for example, a 25-gram subject received a 0.2ml dose regardless of which intervention, route, or species. This required the CC to carefully tabulate the solubility of each compound and the highest concentration possible, if needed to be compatible with the uniform volume acceptable for infusion. In addition to calculating a standard volume that would allow all interventions to go into solution, it is also critical to account for the maximum infusion volume tolerable to the animal. Acceptable and (maximum) tolerable volumes for rodents have been provided below in Supplementary Table 1.

Supplementary Table 1

Acceptable (maximum) daily cumulative volumes in ml/kg body weight

Species	Oral	Subcutaneous	Intraperitoneal	Intramuscular per site	Intravenous (slow injection)
Mouse	10 (50)	10 (40)	20 (80)	0.05 (0.1)	25
Rat	10 (40)	5 (10)	10 (20)	0.1 (0.2)	20

Note. Adapted from "A good practice guide to the administration of substances and removal of blood, including routes and volumes," by Cor Van De Vorstenbosch, Jean-Marc Vidal, David Smith, et a.l, 2001, J. Appl. Toxicol, 21(1), p. 13-23 (https://doi.org/10.1002/jat.727). Copyright © 2001 John Wiley & Sons, Ltd.

To determine how much total drug to fill into each vial, the dose (8µl/gram or 8ml/kg) was multiplied by the largest possible body weight that might be enrolled and then by the number of doses needed per vial. Approximately 20-50% was added to this final volume to account for syringe loss. Knowing the fill and the excipient volume, appropriately sized vials were purchased. Amber vials were used whenever possible in case any of the drugs were light-sensitive (Supplementary Fig. 1a and Supplementary Table 2). Using a standard fill volume, plus overfill, simplified drug bottling at the CC.

Supplementary Table 2

Vial Source and product numbers used.

Model	IV Vials	IP Vials
Mouse ≤ 25 grams	VWR W015271	Life Science 223693
Mouse ≥ 26 grams	Life Science 223693	Wheaton 223738
Rat	Wheaton 223738	EZ Vial 61000G-30

Storage and Stability

Boxes of bottled drugs were shipped at select times during the course of each stage, based on utilization rate and re-stocking needs, and also based on the storage/stability life of each compound had to be evaluated. Some drugs had very short lives after reconstitution, requiring more frequent re-supply. To address this, the CC prepared those limited-stability drugs for shipment by lyophilization for reconstitution immediately before use. A lyophilization/resuspension scheme was devised for each drug based on the physical characteristics of the compound obtained from the manufacturer or inventor. Activity-based bioassays were performed before and

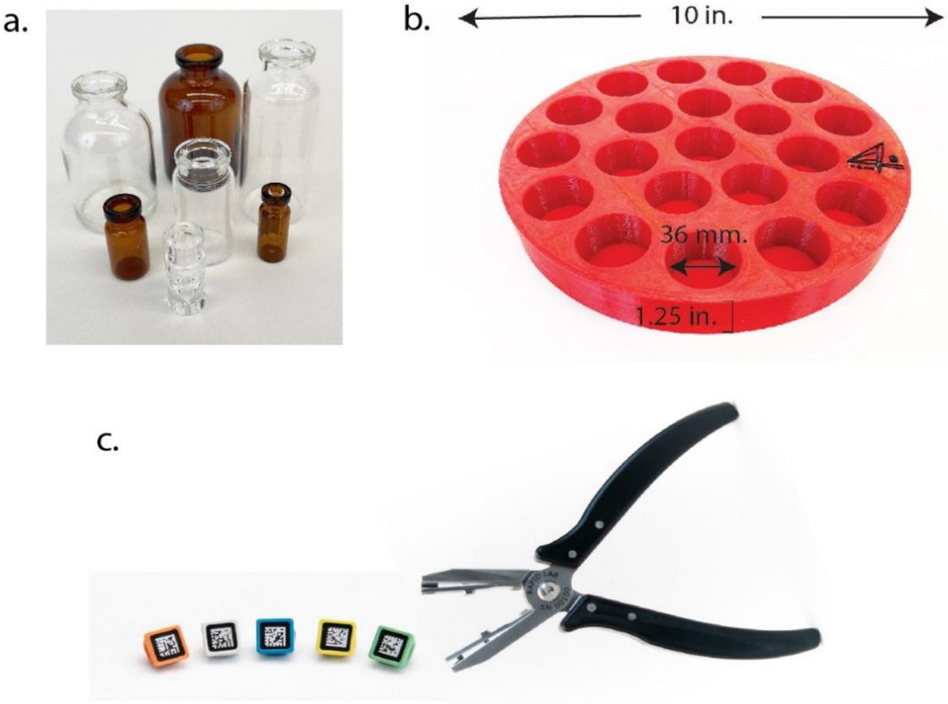

Supplementary Figure 1. SPAN supplies. a, Assortment of vials considered for use in SPAN. b, Custom 3D printed vial trays created to fit the larger vials in the lyophilizer. c, RapID eartags and applicator supplied to all research laboratories.

Supplementary Figure 1

after lyophilization, shipping, and reconstitution to confirm drug activity. To reduce lyophilization volume and avoid 'boil over', the smallest volume possible was lyophilized. For drugs that would be resuspended in water to a final concentration of 0.9% w/v saline, lyophilizing in 5% w/v saline decreased the starting volume considerably. Drugs with longer stability were shipped in liquid, ready-to-use form. These drugs either had activity-based bioassays or HPLC performed to confirm their drug activity after shipment if data was unavailable from the manufacturer.

Drug Preparation, Bottling, Labeling, and Shipping

Drug Lyophilization

Vials used for lyophilization should be extruded type, made from one piece of glass to prevent failure at seams during extreme pressures and temperatures. Stoppers should be non-reactive with drugs and should have appropriate legs/venting for lyophilization. In SPAN, all stoppers were manufactured from an ultra-pure bromobutyl formulation (DWK Life Sciences Wheaton W224100402,

DWK Life Sciences W224100408) and capped with a crimped flip cap (DWK Life Sciences W224205, DWK Life Sciences W224202) to maintain sterility of the septum until use. To facilitate higher throughput drug preparation, custom circular 3D printed trays (10”x 1.25”) were manufactured based on the different vial sizes needed for different stages in SPAN (Supplementary Fig. 1b). Trays were designed to fit the lyophilization chamber (Labconco; 700201000).

Vial Labels

Each filled vial was labeled with a permanent, unique label (Electronic Imaging Materials Electronic Imaging Materials; Stock#562 3 mil Matte White TT Plastic Hot Melt Adhesive 331971-Blue,331971-Green, T68252074). The labels resisted chemicals, water, and freezing. Each label contained the barcode, route, ID, and was color coded according to route as an extra indicator and reminder to the user. Colors were needed that could be seen by investigators with color vision deficiency: blue (Hex #78b3e0) to indicate interventions administered via IP and green (Hex #97d700) to indicate interventions administered IV. Barcode scanners were used to read the vial ID barcodes for data entry. In addition to the label that went around the vial, a small circular label (GA International, www.labTAG.com Catalog # JTTA-53Not; JTTA-53Not and HBCL-61NPNOT) was affixed on the top of the vial with the ID number to facilitate quick identification of assigned vial, thus decreasing the time for the box—and remaining vials—to be out of the refrigerator (Figs. 3a, 3b). These labels were intended to be peeled off and applied directly to the paper surgical record as a part of the raw data.

Box Labels

A labeling convention was designed using letters for boxes and numbers for vials to ensure clarity. The first box shipped to each site was labeled “A”, the second box shipped “B”, and so on. Boxes intended for each research laboratory were labeled with a 2-letter code, e.g., AG for Augusta, UT for the University of Texas- Houston, and so on (Fig. 3b). A 4-digit numbering system for vials was the same across research laboratories to simplify drug packaging and vial box filling. For example, the vial labeled AG546 at Augusta contained the same drug as UT546 at UT-Houston (Fig. 3a). To facilitate drug tracking and quality control, we used the same format for labeling vial boxes across all research laboratories, such that vial box MG-F-IV was the 6th vial box shipped to Massachusetts General Hospital (MG) for IV use in rats and held vials containing the same drugs as UT-F-IV vial box shipped to UT Houston for IV use in mice.

To each box, a pre-printed adhesive label (Chemical resistant Avery paper (AVE60501)) was applied to the top, the front of the lid, and the main front to facilitate the identification and decrease the time out of the refrigerator or freezer (Fig. 3b). Labels were printed on a laser printer using the same color convention used for the vial labels. Storage temperature instructions and compliance text for federal code 21 CFR 312.160 (CAUTION: Contains a new drug for investigational use only in laboratory research animals, or for tests in vitro. Not for use in humans.) were included on the labels.

Vial Box Selection

Freezer-safe cardboard vial boxes with a moisture-repellant coating to prevent warping and disintegration (Grainger TK64538696T) were sized to allow easy insertion and removal of vials,

including caps. In other networks, other containers could be used, with an aim to keep the vials together in a refrigerator or freezer and clearly state the vial ID contents.

Vial Box Organization

We created a standard box-loading scheme across all research laboratories to allow the high-throughput loading of thousands of vials that were blinded to the end user. The pattern should be easy for the person loading the vial boxes, easy to cross-check, and easily repeated across all research laboratories. The pattern of vials loaded in each box was standardized across sites, irrespective of model assignment in each stage. For example. the MG vial for treatment group “B” for rats in a 5 ml vial in box MG-J-IV would be in the same position for group treatment “B” for mice in a 2 ml vial in vial box UT-J-IV (Supplementary Fig. 2). Throughout the trial, animal models varied across the research laboratories and rotated every two to three weeks. Thus, in planning any one shipment, depending on which animal model (mice or rats) might be in use across the research laboratories, vial boxes might be loaded with larger or smaller vials. To maintain uniformity and simplify the vial box loading, the number of vials in each box was fixed across all research laboratories in every shipment.

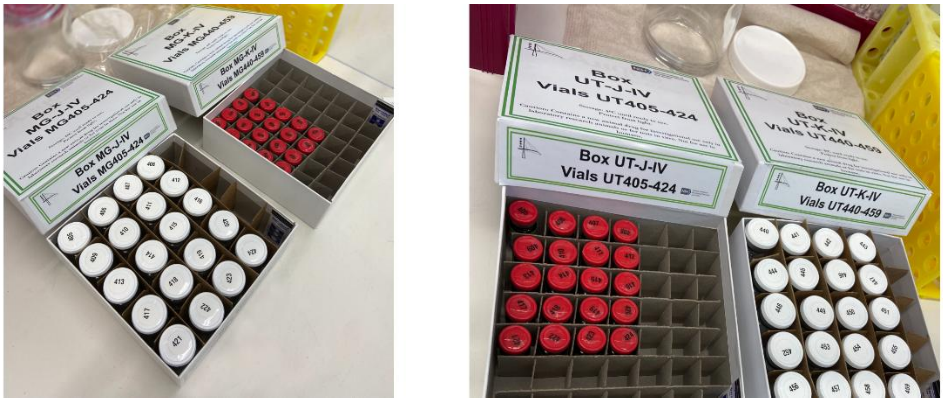

Supplementary Figure 2. Examples of loaded vial boxes J and K ready for shipment to research respective laboratories. Vials in Box J, on the left of each photo, were for use in rats at MG and for mice at UT. Vials in Box K, the vial box on the right of each photo, were for use in mice at MG and rats at UT. Despite the difference between species, each numbered vial contained the same intervention across all sites. For example, Vial MG405 was the same intervention as UT405 even though the vials are different sizes and intended for different species. This pattern of box loading facilitated packaging and helped eliminate errors.

Supplementary Figure 2

Shipping

After loading with the indicated number of vials, boxes were shipped to the research laboratories in Thermosafe Insulated Shipping containers (Sonoco™ ThermoSafe Envoy™ Global Pre-Qualified Insulated Shippers with Summer Packout E3R2S, E6R2S, E12R2S). Temperature indicators (ShockWatch WarmMark: WM 8/46) were placed in each box to monitor for out-of-range temperature shifts. Shipments were scheduled to arrive at the destination laboratories prior to the expiration date of the previous shipment, with allowance for unanticipated delays.

Minimizing Shipping Costs

Over the stages, the process for sending out interventions was refined to minimize cost (Supplementary Fig. 3). In Stage 1, randomization tables and intervention shipments were not aligned with surgery schedules, which resulted in wastage. In subsequent stages, stage specific timelines were developed to anticipate the beginning (Supplementary Table 3) and end of each stage. In later stages, research laboratories were asked to conduct a minimum of 8 surgeries per week so that the randomization tables could be easily aligned with surgery schedules. By distributing these timelines at the beginning of each stage, the CC was able to anticipate inventory and better time shipments to each research laboratory.

	Stage 3 Boxes	Site 1 (OB/AGE)	Site 2 (OB/AGE)	Site 3 (AGE/SHR)	Site 4 (AGE/SHR)	Site 5 (OB/SHR)	Site 6 (OB/SHR)
First Stage 3 Shipment	Box J	E6R2S<br>MICE	E6R2S<br>MICE	E12R2S<br>MICE	E12R2S<br>MICE	E12R2S<br>RAT	E12R2S<br>RAT
	Box K	MICE	MICE	RAT	RAT	MICE	MICE
Second Stage 3 Shipment	Box L	E6R2S<br>MICE	E6R2S<br>MICE	E12R2S<br>MICE	E12R2S<br>MICE	E12R2S<br>RAT	E12R2S<br>RAT
	Box M	MICE	MICE	RAT	RAT	MICE	MICE
Certified Shippers Used	E6R2S (2)	FREEZE<br>(10) FPP2520	REFRIGERATE<br>(4) FPP2510	(LxWxH)	17x16x21 in	48 hr duration	23.4lbs
	E12R2S (4)	FREEZE<br>(20) FPP3010	REFRIGERATE<br>(24) FPP3010	(LxWxH)	21x19x21	48 hr duration	36.1lbs.

Supplementary Figure 3. Sample shipping schedule used in Stage 3. Chart showing the amount of organization required to accommodate the different-sized shipping containers and vial boxes to each research laboratory.

Supplementary Figure 3

Supplementary Note 2

Behavior Ratings and Concealment

Neurological Deficit Score (NDS)

We adapted the standard rating scoring for use in both rats and mice. We defined scores as 0=normal, 1=forelimb and torso turning to one side, 2=circling and circling direction, 3= unable to bear weight on one side, and 4=no spontaneous movement or barrel rolling. NDS scores were recorded at reperfusion, and 24 and 48 hours after MCAo and for Stage 4, at the end of the survival period.

Corner Test

The corner test was adapted from the literature for standardized use at multiple research laboratories[32]. Custom apparatus were built by SPAN investigators. For testing mice, opaque plexiglass boards, 11.8”x 8.7”and 0.2” thick, were fixed at a 30-degree angle with two metal brackets (Fig. 4a). A small opening of ~0.35” was left along the joint at the top of the board and ~0.18” opening at the bottom of the two boards to encourage exploration. For rats, the apparatus was also made with opaque plastic boards, 12” x 18” and 0.2” thick. Testing followed a set protocol at baseline before MCAo and then 7 and 30 days later. The investigator initiated a corner test trial by placing the animal far enough into the corner that both vibrissae touched the walls, stimulating the animal to explore. Each trial ended when the animal made a turn, defined as the head and neck turning > 90° to the body in one direction or the other. The number of right and left turns were counted to a total of 10 turns. A turning index was computed from the number or right turns (R) and left turns (L) using this formula:

$$Abs[\frac{(L-R)}{(L+R)}]$$

Grid Walk Test

Mice were placed onto an elevated platform 1-2 feet from the tabletop with steel wire grids of 16.25” x 12.75” with 1” square openings. Rats were placed onto a platform elevated 2-4 ft from the floor with a steel wire grid of 30” x 30” with 2” square openings. An enclosure was set up around the elevated grid or prevent subjects from exploring the sides (Fig. 4b). Subjects explored the grid until 5 minutes elapsed. Data were expressed as the fraction of foot faults per total number of steps.

Hanging Wire Test

The examiner placed the mouse in the center of an elevated grid (16.25” x 12.75” with 1” square openings) and allowed the animal to spontaneously explore for 5 minutes. The grid was slowly inverted and placed at a height of 7-8 inches above the subject’s home cage, which was bedded to prevent injury. The number of seconds before the animal dropped into the cage, or a maximum of 120 seconds, was scored. This test was repeated for three trials with 20-30 minutes rest between each trial. After Stage 1 it was found that this data added little or no new information, compared to the grid walk test, and was no longer used.

Video Preparation and Blinding

Digital videos of the behavioral tests were recorded at each research laboratory and uploaded to a data repository (Laboratory of Neuroimaging, LONI). The LONI Image and Data Archive (IDA) then relabeled each video with a unique identifier (UID) and created one specific URL link for each recording. Finally, these anonymized video UIDs and video URLs were imported into the correct animal record and correct visit in REDCap (Fig. 4c).

Prior to the start of SPAN, webinars were conducted to instruct the laboratories on SOPs designed to standardize video recording. The recording SOPs were designed to eliminate identifying features and to facilitate scoring. When viewing any single behavior assessment video, the rater did not know the treatment group, post-operative visit day or originating research laboratory.

Data Transfer Security and Interconnectivity

Communication with each research laboratory to troubleshoot and fine-tune the video upload process occurred on multiple levels. Some institutions with more resilient firewalls were not compatible with the IDA software and required institutional approval for software installation prior to the start of SPAN. To eliminate errors during uploading, research laboratories could only upload one video at a time. This reduced the number of potential incorrect uploads to the wrong animal ID.

Video Assignment

Each research laboratory was assigned 30 to 40 video assessments per week. To maintain high throughput, the CC imported video UIDs/URLs from IDA into REDCap daily. Once per week, raters received their assigned UID/URLs on compiled scoresheets that were formatted and locked in Excel spreadsheets. Raters were always only assigned videos from another research laboratory. Raters opened each URL link to view each video and score the behavior on the password protected, locked scoresheet. Behavior scores were sent back to the CC as an Excel.xlsx rather than as a .csv to preserve identifying data hidden from the user with the “hide column” function in Excel. Once received, the ratings were to be returned to the CC within 7 days for import into the correct animal record in the REDCap database (Fig. 4c).

Video Feedback to Research Laboratories

As scores were imported into the REDCap database, quality control checks assured correct subject identifier, and correct rater ID numbers. Queries were generated weekly to correct errors. Raters provided comments on the rated videos to improve video quality, protocol compliance, and serve as data entry verifications. Video links and blinded comments were distributed to research laboratories biweekly in each stage to enhance the quality of the video, protocol compliance, and build a sense of ownership across the laboratories.

An essential part of video protocol compliance was ensuring that the right video recording was uploaded for the correct animal ID at the research laboratory level. To assist with this, the CC requested that research laboratories record the RapID ear tag color in the enrollment form of the REDCap database (Supplementary Fig. 1c). Raters were asked to record the ear tag color seen in the video and if discrepant, the CC would immediately follow up with the recording research laboratory to ensure the correct video was uploaded and that the ear tag color was updated in REDCap. Rater comments also allowed further quality assurance efforts to verify entered scores and pursue any other discrepancies. If comments addressed animal welfare concerns or serious compliance deviations, research laboratories notified the CC immediately.

Supplementary Note 3

Research Laboratory Organization and Best Practices

Research laboratories were provided with stage-specific schedules with included animal ordering and surgery timelines at the beginning of each stage (Supplementary Table 3). From these timelines research laboratories were responsible for developing their own schedules for other tasks required in the stage, e.g., intervention administration, video recording/uploading, MRI assessment/upload, and end of study. Critical scheduling events included behavioral assessments and MRI scanning time. These timelines were based on the experimental protocol approved by the Steering Committee prior to each stage.

See attached Supplementary Table 3 in attached spreadsheet.

In-person site visits were made prior to study start and annually thereafter. Through discussions during the in-person visits, the CC found that research laboratories with the least number of data queries were those who had assigned one investigator for each key role in the workflow. For example, one investigator would be responsible for video recordings and uploads, another investigator would be responsible for surgeries, and another investigator would be responsible for MRI uploads. Research laboratories would share rating responsibilities and assign 2-6 investigators to complete the rater certification for behavior assessments. Research laboratories with the least number of follow up data queries used shared drives such as Box, Google drive, OneDrive, or institution specific servers to assure that source documentation and calendars were accessible to all study personnel. Simultaneously, top performing laboratories stored hard copies of their surgery, video, and MRI notes in a shared paper binder. Best practice included same day (or within 24 hours) update of the online REDCap database so that research laboratory personnel could go back to their records when a data query was initiated and resolve the query quickly. Also, best labs scheduled weekly or bi-weekly dedicated meetings with the local SPAN PI and the entire research team to discuss study progress, suggestions or any encountered in the project.

Randomization and Quality Control

A stage-specific randomization checklist was used to assist the CC in quality checks through the randomization process. An example would look similar to:

- Check that research laboratories have entered equal numbers of male: female ITTs.
- Ensure that the sex of the subject in REDCap matches the line on the randomization table.
- Check that the species and model are correct for the research laboratory and current block.
- Check that model specific details are correct.
- Check that age at the intended surgery date is correct.
- Check that the body weight is within the expected range.
- Check that the surgeon is certified.

To begin the randomization process, the research laboratory submitted an Intention to Treat (ITT) form to the CC indicating the subject identifier and intended surgery date. Certain details (surgery date, surgeon, species, model, weight, age, and sex along with model specific details) are included in the ITT email to the CC that can be used to perform preliminary quality checks. For example, if a research laboratory only submits ITTs for males, then the CC request that they correct by cancelling some of the males and scheduling an equal number of females.

After receiving the ITT form, randomization is completed at the CC and retransmitted to the site at least one working day before the scheduled surgery date. Research laboratories were discouraged from rescheduling surgeries once randomized.

The randomization sequence involved three separate documents to assure accuracy and allow confirmation: the correct research laboratory's randomization table from the Statistics Core, the Master Intervention Scoring Sheet (MISS), and the subject specific randomization form in REDCap. In addition, the process required action from two independent CC staff (referred to as "randomizer" and "verifier") (Supplementary Fig. 4).

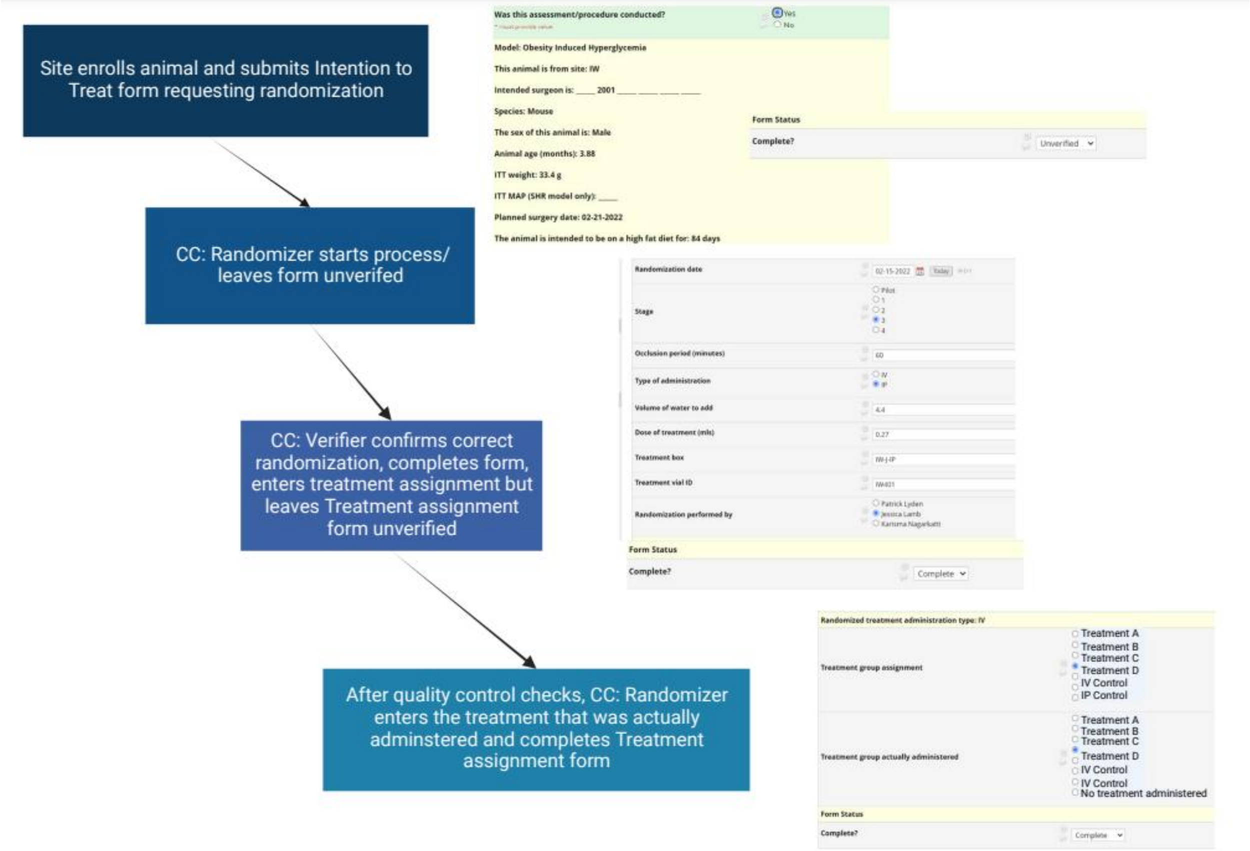

Supplementary Figure 4. A detailed sequential overview of the randomization procedure involving two distinct individuals—the randomizer and the verifier—who independently assign interventions to each subject and subsequently confirm their accuracy. *These independent steps allow for multiple points of quality control.*

Supplementary Figure 4

The MISS document tracks preparation dates, lot numbers, shipment dates and expiration dates for each vial prepared. The sheet automatically converts grams into kilograms, calculates a subject-specific dose and calculates a volume for that prescription. The MISS also serves as the master blinding key: on this spreadsheet the assigned intervention group is stored with the subject identifying information (Supplementary Table 4).

See attached excel spreadsheet for Supplementary Table 4

Animals were randomized using tables generated by the statistical core at the beginning of each stage. The tables were created to stratify by site and sex, and to ensure randomization blocks reflect the workflow and rotation of models. Animals were randomized in sequence and no randomization numbers were re-used if an animal dropped out or was lost to follow up.

The subject-specific randomization form in REDCap was only visible to the CC. Data entered in the randomization form was included in the emailed randomization prescription. Key information needed for randomization was populated into each randomization form from prior forms. Key pre-populated data included model, intended surgeon, species, age, weight, model specific details (e.g., mean arterial pressure or high-fat diet duration) and planned surgery date. The body of the randomization form includes stage-specific details i.e., period of occlusion and the stage of SPAN, in addition to the actual intervention details (vial ID, vial box ID, dose, infusion-rate, mmHg).

To begin, the randomizer confirmed the demographics that were populated in the header of the REDCap randomization form. Then, using the correct randomization table—depending on the sex of the animal and meeting all inclusion criteria per the randomization checklist—the animal ID was entered in the randomization table's next available row and the randomizer read the intervention assignment. The randomizer then entered animal ID and intervention assignment into the MISS at the next available unexpired vial of that assigned intervention along with the animal weight from the REDCap randomization header. Based on conditional formulas programmed using Excel Visual Basic, the MISS then calculated a dose volume and an infusion rate, if given IV (Supplementary Table 5 in the attached Excel spreadsheet). The sex and intended surgery date were also recorded on the MISS document. Next, the randomizer's initials and date were entered, triggering a green highlight on an empty adjacent cell alerting the verifier which rows required quality control checks and verification. Finally, the randomizer entered onto the REDCap research laboratory-specific randomization table: the vial box and ID, stage, length of occlusion, route of administration, dose volume, and rate of infusion if IV. The randomizer signed the form by clicking on their name and marking the form as 'unverified'. The randomizer then locked the REDCap ITT form to prevent research laboratories from changing the surgery dates or making other changes after declaring their 'intention to treat'.

After the REDCap randomization form was marked 'unverified', the verifier checked all data entered on the randomization table, MISS and REDCap randomization form. If the data on the three documents aligned, the verifier entered their initials and date on the research laboratory-specific randomization table and MISS. The verifier changed the status from 'unverified' to complete in REDCap, entered the intervention group assignment on the Treatment/Intervention Assignment form in REDCap and marked this form as 'unverified'. The non-applicable post-operative REDCap forms were locked in 'unverified' status to assist in monitoring users' dashboards and prevent users from entering post-operative data on the wrong form. When the verifier marked the REDCap randomization form 'complete', a randomization prescription was sent via email to the research laboratory, thus completing the randomization process.

Each emailed randomization prescription contained the animal ID and vial ID which were populated from the details entered in the REDCap randomization form and all information needed for the treatment. The email prescriptions were designed to be printed by the research laboratory and used bench side. Research laboratories saved these printed prescriptions (raw data) in binders stored locally and transcribed these details into the appropriate forms in REDCap. These paper copies facilitated later quality assurance audits.

To assess compliance, after surgery was completed, the CC compared vial IDs entered on to the Randomization form and the Surgery Day form to verify that the assigned vial was given to the subject. Once the intervention administration was confirmed, the CC entered the intervention on the Treatment/Intervention Assignment form in REDCap and changed the status from ‘unverified’ to ‘complete’.
